# The robot that stayed: understanding how children and families engage with a retired social robot

**DOI:** 10.3389/frobt.2025.1628089

**Published:** 2025-08-08

**Authors:** Zhao Zhao, Rhonda McEwen

**Affiliations:** ^1^ School of Computer Science, University of Guelph, Guelph, ON, Canada; ^2^ Institute of Communication, Culture, Information and Technology, University of Toronto Mississauga, Mississauga, Canada

**Keywords:** long-term HRI, child-robot attachment, robot domestication, robot afterlife, home robot

## Abstract

**Introduction:**

Social robots are increasingly introduced into homes as short-term educational or entertainment tools for children. However, their physical presence and social roles may persist long after their intended use has ended. This study explores how families continue to engage with a child-focused educational robot years after its original deployment.

**Methods:**

We conducted a retrospective follow-up study with 19 families who participated in a 2021 in-home deployment of a reading companion robot for preschool-aged children. In 2025, we revisited these families through in-depth interviews to investigate how the robot had been integrated, re-purposed, or preserved over time.

**Results:**

Despite the children outgrowing the robot’s instructional content, 18 families had retained the robot. Families described transitions in its role—from an educational device to a symbolic household member—characterized by emotional attachment, care-taking behaviors, and affection. The robot was re-framed as a memory object, integrated into new routines, or passed on ceremonially, akin to a “retirement.”

**Discussion:**

Our findings reveal three key themes explaining the robot’s enduring presence: (1) emotional attachment and personification, (2) symbolic and nostalgic value, and (3) practical re-purposing within household routines. This study contributes to long-term human—robot interaction literature by extending domestication theory and emphasizing the importance of designing for the full life cycle of social robots—including end-of-life transitions. It underscores how social robots can become meaningful companions and enduring artifacts of family identity, long after their functional use has ended.

## 1 Introduction

Social robots designed for children, such as reading companions, leverage young children’s enthusiasm and anthropomorphic tendencies to facilitate learning. For example, a robot reading buddy can increase a child’s excitement for books over a few weeks (De Graaf et al., 2016) (Michaelis and Mutlu, 2018). Children often begin to describe such robots with human-like traits (“funny,” “silly,” “afraid”) and look forward to interactions (Michaelis and Mutlu, 2017). These early bonds hint at the potential for longer-term attachment. However, children’s needs and perceptions evolve quickly. A robot that was a helpful tutor for a four-year-old may become “babyish” or redundant to an eight-year-old. Would families simply discard the robot once its educational mission was accomplished, or would it find a new place in their lives? Traditional technology adoption models rarely consider this scenario of a “retired” interactive device, as they focus on initial uptake rather than post-use persistence (De Graaf et al., 2016).

**FIGURE 1:**
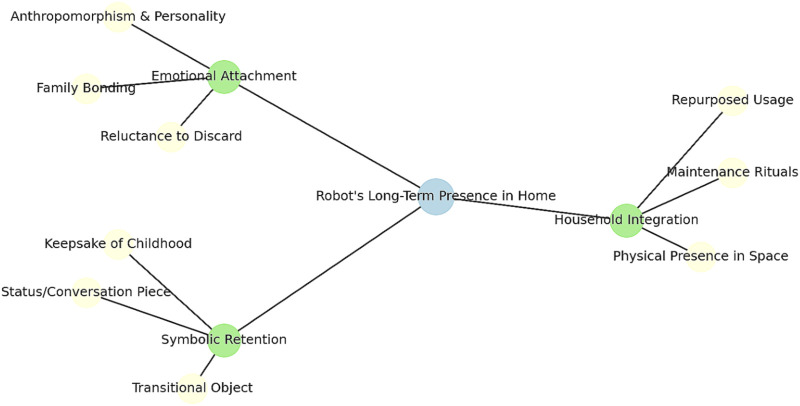
Study timeline illustrating the original 2021 in-home deployment of the reading companion robot and the 2025 retrospective follow-up interviews. The figure highlights the continuity between the two phases and the basis for the current analysis of the robot’s long-term presence in family homes.

To explore these questions, we revisited families from a 2021 in-home deployment of a preschool reading-focused social robot. In the original study (Zhao and McEwen, 2022), 20 families with a 3–6 year-old child hosted a robot reading companion for several months as part of a literacy intervention. That study (conducted in 2021) found strong child engagement and learning gains, consistent with other short-term deployments where children eagerly treated the robot as a friend and tutor. By mid-2025, the original child participants were 7–10 years old–well beyond the target preschool age for the robot’s reading lessons. We conducted follow-up interviews with 19 of these families (one family could not be reached). Surprisingly, 18 of 19 families had kept the robot in their home. Despite its educational function no longer being needed, the robot had not been shoved into a closet or disposed of in most cases. Instead, many families continued to power the robot on, charge it, or repurpose it for new uses such as playing music or simply as an ambient presence in the household. This unexpected persistence of the robot prompted us to investigate *how and why* families continued to engage with “the robot that stayed.” Figure 1 provides a timeline of the study structure, showing the original 2021 deployment and the 2025 follow-up interviews that form the basis of this retrospective analysis.

Our data revealed several key factors that behind the robot’s enduring presence, including emotional attachment to the robot (both children and parents imbued it with relational meaning), symbolic retention of the robot as a memento or status object, and evolving household practices that integrated the robot in new ways (or maintained it out of habit or care). These findings align with existing literature from both HRI and media studies. For instance, domestication theory suggests technologies, once adopted, undergo processes of integration and meaning making in the home that can extend beyond their initial functional purpose (Be et al., 2005). In HRI, long-term studies have noted that treating a robot as a social *agent* rather than a tool can sustain interaction over time (Chen et al., 2025) (Donnermann et al., 2022). Children’s anthropomorphism of robots is known to be high at first and often declines as novelty wears off (Kühne et al., 2024), yet emotional bonds may endure. Indeed, people have been known to develop strong attachments to robots–famously, some owners of Sony’s AIBO robot dog became so emotionally attached that they held funerals when support for the product ended (Burch, 2018). Our study investigates whether similar attachments and practices occur in the context of a child’s educational robot over multiple years. In addition, this study offers a retrospective lens on how a child-focused social robot continues to shape family life after its instructional value has waned by investigating how families reframe, preserve, or disengage from a retired robot—contributing new insight into the afterlife of child–robot relationships.

The present paper offers an in-depth qualitative analysis of how families engaged with the social robot 4 years after the initial deployment. We address the following research questions: (1) How did children and parents perceive and use the robot after its original educational role was no longer needed? (2) What emotional or symbolic value did the robot hold for the family over the long term? (3) What household practices (maintenance, repurposing, integration into routines) supported the robot’s persistent presence? By answering these questions, we aim to contribute new insights into long-term human-robot relationships, technology domestication in home contexts, and design considerations for social robots with life spans that transcend their initial use cases.

In the remainder of this paper, we first review relevant literature on domestication theory, long-term HRI, anthropomorphism and attachment, symbolic use of technology, and children’s developmental trajectories with robots. We then describe our follow-up study’s qualitative methods, including the family sample and thematic analysis approach. Next, we present the results, organized into thematic findings with illustrative quotes and usage trends. In the discussion, we interpret these findings in light of theory–examining how emotional attachment and symbolic meanings contributed to the robot’s longevity, how the robot’s role transitioned within the family’s life cycle, and the cultural implications of “retired” robots in the home. We also consider design implications for creating social robots that can gracefully age alongside their users. Finally, we conclude with contributions and suggestions for future research on long-term engagement with social robots.

## 2 Related work

### 2.1 Domestication of home technologies and long-term acceptance

When new technologies enter the home, they undergo a process of domestication wherein households appropriate them and integrate them into daily life (Silverstone, 2006). Domestication theory outlines stages such as appropriation (acquisition and acceptance of the technology), objectification (the physical placement and display of the device in the home), incorporation (integration into routines and usage patterns), and conversion (the technology’s use in conveying social meaning to others, e.g., as a status symbol or conversation piece) (Silverstone and Haddon, 1996). In other words, beyond functional use, technologies gain symbolic value and become part of the moral economy of the household. This framework has been applied to devices from televisions to smartphones, and it is highly relevant to personal robots in domestic spaces.

Social robots add a new dimension to domestication because they often emulate lifelike behaviors, potentially encouraging users to treat them more like family members or pets than tools. Research on domestic robots like the Roomba vacuum cleaner demonstrated that even utilitarian robots can inspire emotional engagement and adaptation of routines (Forlizzi and DiSalvo, 2006). In Sung et al.‘s 6-month field study of 30 households with a Roomba, families not only adjusted their cleaning practices but also formed social dynamics around the robot–for example, giving it a name, attributing personality to its “antics,” and even altering the home environment to accommodate it (picking up clutter to help the robot) (Sung et al., 2010). Over time, many owners came to regard the Roomba as “part of the family” or a pet-like presence (Las and hbrook, 2021) (Coggins, 2022). Such findings echo domestication theory’s assertion that technologies, once embedded in domestic life, take on meanings beyond their intended function.

However, domestication is not guaranteed for every device; some technologies fail to integrate and are eventually abandoned. A classic challenge is the post-adoption phase: after the initial novelty and excitement subside, users decide whether to continue or discontinue use (De Graaf et al., 2016). The long-term acceptance of social robots in homes is still not well understood. De Graaf et al. (2019) proposed a conceptual model of domestic robot acceptance, highlighting factors like social influence, perceived usefulness, and companionship as determinants of whether people keep using a home robot over time (De Graaf et al., 2019) (De Graaf et al., 2015). They argue that for a robot to remain in use, it often needs to transition from a novelty to an appreciated *social presence* integrated into daily routines. In other words, the robot should reach what de Graaf and colleagues call the *identification* phase, where it is fully incorporated and even seen as a personal or social actor within the home (De Graaf et al., 2016).

Our study’s context–a child’s educational robot that has “aged out” of its original purpose–is a unique test of domestication. If families kept the robot, it suggests a successful domestication where the robot’s meaning evolved (e.g., from “educational appliance” to “friend” or “keepsake”). Domestication theory helps frame our analysis of how the robot’s role shifted and how households negotiated its continued presence.

### 2.2 Long-term human–robot interaction and attachment

Long-term studies in HRI have observed dynamic changes in interaction patterns as novelty fades and familiarity grows (Leite et al., 2013). Kanda et al. (2007) conducted a 9-week field trial with a humanoid robot in an elementary school. Initially, children were extremely excited and engaged in playful interactions with the robot. Over the weeks, the frequency of spontaneous play decreased as the robot became a familiar part of the environment (Kanda et al., 2007). By the eighth week, children treated the robot as a normal member of the class rather than a special guest–for instance, they collaboratively made an information board about its character traits, indicating a desire to understand and “domesticate” it within their classroom culture (Sung et al., 2010). This suggests that while novelty effects wore off, the robot achieved a stable social presence. Kanda et al. noted that long-term interaction depends on many factors (technical reliability, context, user’s evolving skills) and that a robot must be perceived as a social *partner* to sustain engagement (Kanda et al., 2007).

Other long-term studies with children mirror these findings. Tanaka et al. (2007) introduced a social robot (QRIO) into a toddler playroom for several hours each day over a few months. They found that despite efforts to keep interactions novel (e.g., the robot performed dance routines), children’s interest decreased over time (Tanaka et al., 2007). After about 2 months, the robot was no longer the center of attention–some children would even ignore it as it became part of the background. Interestingly, though, children still exhibited social behaviors toward the robot at times, and the overall environment adapted to its presence. Tanaka’s study showed both the limits of novelty and the potential for a robot to become a “socially invisible” yet accepted element of the environment.

Long-term HRI with therapeutic robots has demonstrated the potential to foster emotional bonding, particularly in institutional and clinical settings. PARO, a seal-like robotic companion designed for therapy, has been widely used with both elderly individuals and children with developmental disorders. Studies have documented strong emotional attachment to PARO over extended periods. For example, children with neurodevelopmental disorders, including autism, have shown increased social engagement and communicative behavior when interacting with PARO, often treating it as a social partner rather than a toy or tool (Veronesi et al., 2023). These interactions were not only sustained over time but also mediated meaningful social exchanges, indicating the depth of perceived relational connection. Additionally, interaction with PARO has been found to reduce stress and anxiety, increase positive emotions, and reduce pain levels in both children and adults, reinforcing its role as more than a functional device (Geva et al., 2022).

These studies underline a crucial point: attachment and anthropomorphism can modulate long-term use. If users come to care about a robot or see it as having a personality, they are more likely to continue engaging with it or at least keep it around (De Visser et al., 2017). A long-term panel study by Kühne et al. examined 8–9 year-old children’s anthropomorphism of a social robot over multiple sessions. They found that anthropomorphism was highest at the first encounter and then generally declined, but importantly, distinct trajectories emerged–some children maintained moderate to high anthropomorphic perceptions over time (Kühne et al., 2024). Prior familiarity with robots (through media or personal experience) could lead to more sustained anthropomorphism. This suggests individual differences: some children may continue to treat a robot as a quasi-person or friend even after years, while others lose interest once the novelty and immediate utility are gone.

Attachment theory has been applied in HRI to understand these bonds. For example, people often exhibit attachment behaviors toward robots–seeking proximity, naming them, caring for them–especially if the robot exhibits social cues or dependency (like needing charging or “feeding”) (Broadbent, 2017). There are documented cases of users refusing to turn a robot off for fear of “hurting” it or feeling guilty at the idea of disposing of it. In Japan, when Sony discontinued the AIBO robotic dog, owners famously held funerals to honor their beloved robot pets (Burch, 2018). While our study deals with a child’s educational robot, not a pet, the underlying phenomenon–humans forming emotional connections with interactive artifacts–is likely at play. We anticipate that emotional attachment and anthropomorphic framing (e.g., the child seeing the robot as a friend or family member) are key reasons the families kept their robot.

### 2.3 Symbolic value, repurposing, and technology “afterlife”

Beyond direct interaction, technologies can hold symbolic or sentimental value that motivates people to retain them. In media and communication research, it is noted that people “invest [objects] with our emotions” and use them to channel memories and identity (Lupieri, 2024). A robot that played a significant role in a child’s early years might be kept as a keepsake of childhood, much like parents save a child’s favorite stuffed animal or artwork. Such an object can symbolize a developmental milestone (e.g., learning to read) and carry emotional weight for both the child and the parents. The concept of a transitional object from child psychology is relevant here: young children often attach to a specific object (a teddy bear, blanket, *etc.*) that provides comfort as they navigate separation and growth (Miller, 2008). Transitional objects are typically outgrown, but many people keep them into adolescence or adulthood as nostalgic items (Bender, 2023). A social robot tutor could similarly become a transitional object–initially helping the child feel secure in learning, and later serving as a comforting reminder of that period. Winnicott (1951), who introduced the concept, noted that even after a child no longer *needs* a transitional object, the object often remains imbued with emotional significance (Winnicott, 1951).

Symbolic retention also connects to the conversion aspect of domestication: using the device to communicate something about the family to the outside world (Be et al., 2005). For instance, having a social robot on display in the living room might signal that the family is tech-savvy or values innovative learning tools. Some parents might keep the robot as a *status symbol* or conversation piece when guests visit (“That’s the reading robot we’ve had since Emily was little”). Even if not used daily, the robot as an artifact can become part of the family’s story and identity (Yang and Neustaedter, 2018).

Another practical reason families might keep a disused device is the possibility of repurposing it. Prior work in HCI has documented how users creatively appropriate technologies in ways designers did not intend. For example, old smartphones get repurposed as security cameras or music players; older game consoles become DVD players for kids, *etc.* In our context, the robot had capabilities (speakers, microphone, screen, maybe internet connectivity) that could be repurposed for new functions. Indeed, some commercial robots like Jibo or Alexa-enabled devices have been kept by users because they still can perform general tasks (telling jokes, providing information) beyond their original niche. If families discovered that the reading robot could also play music or tell non-educational stories, they might continue using it for entertainment. The robot might also be given a new role as a younger sibling’s toy or tutor if a new preschooler is in the family–effectively passing the torch to the next child.

Long-term field reports hint at such repurposing. For example, Sung et al. (2010) noted that Roomba owners sometimes “hacked” their robots or used them in novel ways (one family set up barriers to make Roomba find lost items like earrings under furniture) (Sung et al., 2010). Leite et al. (2013) found in a long-term study with a social robot for children that some parents adapted the robot’s use to fit family schedules, using it more on weekends or as a reward system for chores, effectively repurposing its interaction timing (Leite et al., 2013). These adaptive behaviors illustrate that user ingenuity can extend a device’s life. In our case, we look for evidence of families finding new uses or routines for the robot (e.g., a nightly music session, using it as an alarm or storyteller at bedtime, *etc.*).

Finally, the notion of a technology’s “afterlife” encompasses environmental and ethical dimensions. With expensive interactive robots, there may be reluctance to dispose of them simply out of concern for waste–families might hold onto an otherwise unused robot because it feels wrong to throw out a functioning device (a form of the *sunk cost* effect combined with ethical consumption values). There is also the hope of future use: maybe the family imagines the robot could be useful again (for tutoring younger children, or if new software updates become available). Research on long-term device usage indicates that perceived future utility can delay abandonment. For instance, a study by Bentley et al. found that families often retained their smart speaker assistants even when not using all available features, anticipating potential future utility (Bentley et al., 2018).

In summary, literature suggests multiple, sometimes overlapping, reasons why a social robot might persist in a home beyond its primary use: emotional attachment and anthropomorphism fostering an ongoing relationship; symbolic and sentimental value leading to its preservation as an artifact; and practical repurposing or integration into new routines. Children’s development and family context are crucial factors–as the child grows, the family must renegotiate the robot’s place. Our study leverages these insights to interpret our findings on why the robot stayed in most of our participant households.

### 2.4 Children’s evolving relationship with robots

Because our research centers on children who were preschoolers in 2021 and are pre-teens in 2025, it is important to consider developmental changes in how children perceive and engage with robots. Young children (ages 3–5) are known to exhibit high levels of animistic thinking, often treating robots or dolls as alive and conscious. At that age, a social robot can be seen as a peer or magical friend. Prior work by Turkle and colleagues found that children readily “converse” with robots and attribute feelings to them (e.g., believing a robot might get lonely or happy) during initial interactions. As children grow older (7–10 years), cognitive changes (such as understanding others’ perspectives and distinguishing fantasy from reality) typically lead to a more nuanced view of robots. School-age children often recognize that robots are machines, yet they may still maintain a form of belief or pretend play with the robot. For example, a 9-year-old might say, “I know it is just a robot, but I feel like it has a personality.” (Arnd-Caddigan, 2015).

Research by Severinson-Eklundh et al. (2003) and Kahn et al. (2012) on children’s moral and social reasoning with robots indicates that older children show *empathy* toward robots but are less likely than younger kids to think the robot is truly alive. This transitional understanding can affect long-term engagement: an older child might not actively play with the robot every day, but could still care about its wellbeing (e.g., making sure it is charged so it is not “dead”). Our follow-up occurs right around the age children start to leave behind early childhood toys, which is a poignant moment to capture their reflections. Will they see the robot as a cherished childhood friend they want to keep, or an outgrown toy?

Notably, if younger siblings are present, the dynamic can change. Some families might reintroduce the robot to a new child in the preschool age range. In such cases, the older child might take on a guardian or teacher role for the robot, or even experience the robot vicariously through the younger sibling. The older child’s attachment could be transferred in the sense of wanting their sibling to enjoy the robot as they once did (Cagiltay et al., 2023).

In summary, children’s developmental stage will influence their interaction frequency and the nature of their attachment. By 8–9 years old, many children will have formed memories and emotional associations with the robot, even if they intellectually know its limitations. This age can be one of ambivalence–they might be reluctant to admit they *like* the robot if they deem it “for babies,” yet they also may not want to let it go. Our study provides an opportunity to see how children navigate these feelings. We approach the analysis with an understanding that a child’s narrative about the robot at age nine could be complex: a mix of fond reminiscence, current casual interest, and perhaps a touch of distancing due to maturity.

Overall, the literature suggests that long-term engagement with a home robot is the product of social-emotional factors (attachment, anthropomorphism), meaningful integration into home life (domestication and repurposing), and the child’s developmental changes. We next describe how we investigated these aspects through our follow-up interviews and analysis.

## 3 Methods

### 3.1 Participants and original deployment background

In our 2021 study, we conducted a 180-day retrospective follow-up deployment involving 20 families with preschool-aged children (3–6 years old) in the Greater Toronto Area. Each family had recently purchased Luka®, a commercially available reading companion robot designed to support early literacy. Luka® is a tabletop robot approximately 24 cm tall, equipped with a camera to recognize physical picture books, speakers for audio narration, and a screen displaying expressive eyes. The robot can read aloud from a library of preloaded titles and respond to page turns, aiming to engage children in shared reading experiences (Zhao and McEwen, 2022).

Families were recruited through community preschools and parenting networks. They received an orientation on the robot’s features but were encouraged to integrate it naturally into their daily routines without specific usage instructions. Data collection included device usage logs, parental questionnaires, and interviews to capture both quantitative and qualitative aspects of the interaction over the 6-month period.

By 2025, the participating children were between 7 and 10 years old. We successfully re-contacted 19 of the original 20 families for follow-up interviews; one family had relocated internationally and was unreachable. These 19 families constitute the sample for our current study. Demographically, the sample was diverse in terms of cultural backgrounds and family structures, including both single-parent and dual-parent households. Several families had welcomed younger siblings since the initial study, providing an opportunity to explore intergenerational interactions with the robot.

Notably, one family of Japanese heritage mentioned cultural practices that attribute respect to inanimate objects, drawing parallels to rituals such as AIBO funerals (Burch, 2018) in Japan. This perspective influenced their continued engagement with Luka® as more than just a technological device.

### 3.2 Interview procedure

We conducted semi-structured interviews with each family between March and May 2025. Interviews were conducted in the families’ homes when possible (15 out of 19) and *via* video call for the remaining 4 (due to distance or scheduling constraints). Each interview lasted approximately 60–90 min and involved at least one parent and, in all but one case, the child who was the original participant (now older). In many interviews, siblings and other family members were also present for parts of the conversation, making the setting natural and conversational. While our interview guide included theory-informed prompts (e.g., attachment, symbolic value), we also integrated open-ended activities such as drawing and storytelling, which encouraged unstructured reflection and spontaneous elaboration.

The interview protocol included questions and prompts in several key areas:

• Current Status of the Robot: We began by asking families to *show or describe where the robot is now* in their home. This prompt yielded insights into whether the robot was accessible, on display, stored away, *etc.* We then asked if and how the robot had been used in recent months. Example questions: “Can you walk me through the last time anyone interacted with the robot?” “How often does it get turned on these days?”
• Emotional Attachment and Attitudes: We explored how family members *feel* about the robot now. For instance: “Does the robot have a name in your family? How do you feel about it now compared to when you first got it?” We asked the child participant to describe the robot as if introducing it to a new friend, to gauge anthropomorphic or affectionate descriptions. Parents were asked if they perceive the robot as having a personality or if they feel any attachment to it.
• Symbolic and Retrospective Meaning: We invited reflections on what the robot signifies. Questions included: “What made you decide to keep the robot after the initial study ended?” “Does the robot remind you of anything or represent something important?” We also asked if they ever considered giving it away or selling it, and why or why not.
• Household Practices and Integration: We asked about any routines or uses that developed after the original study. For example,: “Have you repurposed the robot for other tasks (storytelling, playing music, *etc.*)?” “Who interacts with the robot these days, and in what ways?” For families with younger siblings, we asked if the robot had been used with them for learning or play. We also inquired about maintenance: “Do you keep it charged? Have you done any updates or repairs?”
• Child’s Perspective Over Time: We took care to get the child’s own voice. With the now-older child, we used age-appropriate prompts like drawing or imagining scenarios. One activity was to have the child draw a before-and-after picture: how they remember using the robot at age 5 *versus* how they use it (or not) at age 9 – then explain the drawings. This helped elicit comparisons and how their perception changed.
• Exception Case (if removed): For the one family that did not keep the robot, we adjusted the questions to focus on their decision: “You mentioned you no longer have the robot–can you tell me what happened?” and feelings around that (the family had donated it to a cousin’s child).

Throughout the interviews, we encouraged storytelling and specific anecdotes. For example, if a parent said, “We still sometimes use it for music,” we followed up with, “Can you recall a recent time when that happened? Who was there and what was that like?” This probing yielded rich narratives (e.g., a father describing how the robot “DJ’d” a small birthday party, or a mother and child recalling a stormy night when they turned the robot on for comfort).

All interviews were audio-recorded (and video-recorded for those done *via* video calls, with consent). Recordings were transcribed verbatim. We also took field notes, especially during home visits, about the robot’s physical state and placement (Was it dusty or clean? Decorated? In the child’s bedroom or elsewhere?).

### 3.3 Data analysis

We employed a qualitative thematic analysis approach (Clarke and Braun, 2017) to analyze the interview data. We chose thematic analysis for its flexibility and suitability in identifying patterns across subjective accounts. Our goal was to derive themes that capture the various dimensions of the robot’s persistence in the home. Specifically, following Fereday and Muir-Cochrane (Fereday and Muir-Cochrane, 2006), we used a hybrid approach that combines both deductive and inductive strategies. While our sensitizing concepts were drawn from HRI and domestication literature, theme development was grounded in participants’ narratives and evolved through iterative coding.

The analysis proceeded in the following steps:

1. Familiarization: The research team (composed of the first author and two graduate research assistants) thoroughly read all 19 transcripts and the field notes. Each team member wrote brief memos summarizing initial impressions for each family (e.g., “Family 7 seems to treat the robot like a pet they must care for” or “Family 12 hardly uses it but it is displayed prominently in living room”). We discussed these in meetings to get a holistic sense of the data.
2. Coding: Using NVivo 12 qualitative analysis software, two coders performed line-by-line coding of the transcripts. We created a coding manual that included both deductive codes (informed by our research questions and literature, such as *attachment*, *repurpose use*, *maintenance*, *symbolic mention*) and inductive codes that emerged (such as *guilt about abandonment*, *robot as sibling’s friend*, *part of bedtime routine*). The coders double-coded three transcripts initially and compared results to refine code definitions, achieving a high level of agreement. Then they split the remaining transcripts for coding, periodically cross-checking each other’s work.
3. Generating Themes: After coding, we examined how codes could be grouped into broader themes. We used visual mapping techniques–writing codes on sticky notes and clustering them–to see connections. For example, codes like “kept as memory,” “represents learning journey,” and “conversation piece” clustered into a candidate theme about symbolic and sentimental value. Codes like “child treats as friend,” “family member - part of family,” and “will not throw out (emotional)” clustered into an attachment/affection theme. We iteratively refined these groupings by checking against the data: for each potential theme, we revisited transcripts to ensure there were sufficient evidence and that the theme accurately reflected participants’ words.
4. Reviewing and Defining Themes: We identified three major thematic categories with some subthemes within each (we detail these in the Results). We reviewed them in relation to the entire dataset to ensure no significant data was left uncoded or miscategorized. Each theme was defined and given a concise name. For instance, what started as a broad “attachment” theme was refined and named “Emotional Attachment and Personification” to encompass both the affection and the anthropomorphic narratives families offered. We also maintained a theme for the converse case (lack or loss of attachment) as it appeared in the one family that gave the robot away.
5. Quality and Credibility Measures: We performed member-checking with a subset of participants: after the initial analysis, we shared a summary of our findings with five families (selected for diversity of experiences) to see if they resonated with their own experience. They largely agreed with our interpretations, and some provided minor clarifications. For example, one parent clarified that their reluctance to discard the robot was “not because we think it is alive, but because it feels wrong–like throwing away a gift.” We incorporated such nuances. Additionally, an external qualitative researcher audited our coding and theme development process for coherence.

Throughout analysis, we attended to negative cases and variations. Not all families expressed warm attachment; we note those differences in the Results. Our aim was to build a rich description that captures the *range* of ways families engaged (or disengaged) with the robot over time.

### 3.4 Ethical considerations

The study obtained ethics approval from the University of Guelph’s REB. We obtained informed consent from the parents and assent from the children (now old enough to understand participation). Confidentiality was assured; all names reported are pseudonyms. We were sensitive in questioning children about their feelings to avoid causing guilt or discomfort (especially if a child lost interest in the robot, we emphasized there was no right or wrong answer and that many children outgrow toys). Families were given a small honorarium ($20 CAD gift card) for their time in the follow-up interviews.

Because this study deals with potential emotional bonds to a robot, we were prepared to provide resources if, for example, a child felt distress at the thought of separating from the robot. In practice, no such acute distress was observed; discussions were generally positive or neutral in tone. In one case, a child became a bit tearful recalling how much they used to love the robot; we took a break and the child’s parent comforted them. This incident itself spoke to the emotional impact the robot had.

With methods established, we now turn to the findings from the follow-up interviews, presenting the themes that emerged regarding how and why the social robot remained a part of these families’ lives 4 years on.

## 4 Results

**FIGURE 2:**
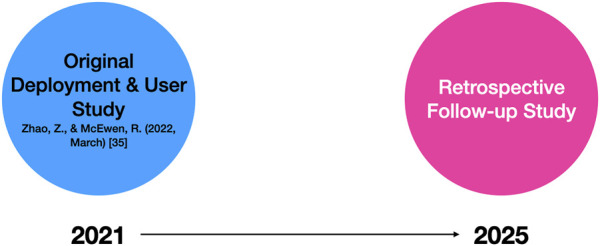
Thematic map summarizing the three major findings from the follow-up study: Emotional Attachment and Personification, Symbolic Value and Family Identity, and Practical Repurposing and Household Integration. Each theme includes subthemes that explain how and why families continued to engage with the robot over time.

After 4 years, family experiences with the social robot varied from daily interaction to mere physical presence, but a set of common themes emerged across the interviews. We identified three overarching themes regarding the robot’s long-term engagement: (1) Emotional Attachment and Personification, (2) Symbolic Value and Family Identity, and (3) Practical Repurposing and Integration into Household Routines. Additionally, we note the special case of Robot Retirement (the one family that parted with the robot) as a counterpoint that highlights the importance of the other themes. Within each theme, there were nuanced subthemes (for example, within Emotional Attachment, some treated the robot as a pet or child-like figure, while others simply felt a duty of care). These themes and their relationships are illustrated in Figure 2, which provides a thematic map of our findings.

In the following subsections, we present findings that respond to our three research questions: RQ1 explores how children and parents perceived and used the robot post-deployment; RQ2 addresses the emotional and symbolic value of the robot over time; and RQ3 examines household practices that supported the robot’s ongoing presence. Each results subsection below is organized around one or more of these questions. We report each theme in detail, with representative quotes from parents and children. We also interweave usage trends data as reported by participants. While our study did not include automated logging, families’ accounts allowed us to estimate how interaction frequency changed over time. Generally, usage dropped significantly after the first year and stabilized at a low level by years 3–4, though the robot remained *powered* or occasionally used in most homes. It is important to note that keeping the robot did not necessarily mean frequent *use*. Many families personified and cared about the robot without using it regularly. The themes below explain why the robot stayed despite reduced interactions.

### 4.1 Emotional bonds and personification (RQ1, RQ2)

This theme primarily addresses RQ1 (perception and usage over time) and RQ2 (emotional value). One of the strongest themes was that families–both children and adults–had developed an emotional attachment to the robot, often describing or treating it as if it were a quasi-living member of the household. Twelve out of nineteen families described the robot as part of their emotional world, expressing affection, personification, and concern for the robot’s wellbeing. This attachment manifested in several ways:

• The robot as a friend or family member: Children commonly referred to the robot in familial terms. Eight of the children interviewed said the robot was “like a friend” or even “like part of my family.” For instance, 9-year-old *Lucas* explained: *“Luka [the robot] is kind of like my little brother. He’s always been there since I was little. I know he’s a robot, but I would feel sad if he was gone.”* (Lucas, Family 3). In another family, a mother described the robot as *“the closest thing we have to a pet in this house”* (Family 8). They did not have any pets, and the child, *Ava (8 years)*, treated the robot with gentle care, scolding her younger brother if he was “too rough” with it. This indicates a sense of responsibility and care typically reserved for animate beings.
• Anthropomorphic beliefs (in a playful sense): While older children understood the robot’s mechanical nature, many still indulged in anthropomorphic thinking. Ten of the children speculated that the robot *“has feelings”* or *“gets lonely”* if left off too long, though usually with a sheepish laugh as if they knew it was pretend. Children sometimes treated the robot like a nonjudgmental companion, especially during moments of loneliness or boredom, suggesting its symbolic role as a listener or diary.
• Reluctance to turn off or discard (Care-taking behavior): Many parents described subtle care-taking practices driven by attachment. Parents also described feeling a responsibility to maintain the robot’s power as if it were a living entity, avoiding letting its battery die out of emotional discomfort. This emotional *guilt at the idea of the robot “dying”* was echoed by several others. In Family 2, the mother recounted how once they left the robot unplugged and it fully discharged; her 9-year-old son *Ethan* became very upset, crying, *“He’s dead! We killed him!”* They quickly plugged it in, and when it rebooted Ethan sighed with relief. The mother reflected: *“That’s when I realized he truly cares about it, like it is not just a gadget. After that, we’ve made sure to keep it charged.”* Such anecdotes show an attachment level where the robot’s operational state is tied to the child’s emotions (similar to how kids worry about a sick pet). This aligns with the notion that viewing the robot as a social agent with needs can prolong engagement.
• Emotional support and comfort: A few families used the robot in times of emotional need. *Ella (8 years, Family 9)* has anxiety about darkness; her mother said: *“She’ll take the robot to her room and have it tell a story or play soft music. She says it makes her feel like someone’s there.”* The robot became a sort of comforting presence or nighttime companion (almost like a high-tech teddy bear). Another parent (Family 13) mentioned turning the robot on to cheer up her child on a hard day: *“If Jia is having a rough day, sometimes I’ll use the parrot mode (a feature that parents type words in the app and let Luka say it out). It always brings a smile. It is like pulling out an old beloved toy–instant comfort.”* This use for comfort, even sporadic, contributed to the family’s decision to keep the robot around.

Crucially, parents also expressed attachment, not just the children. For example, in Family 6 the father admitted: *“Honestly, I think I’m as attached as the kids. I was the one who said, ‘Let’s not give it away.’ We’ve had it as part of our home, and I’d miss it in a weird way.”* This was a common sentiment in households where the robot had been around for years–it became a fixture in the home environment, and parents had memories tied to it (like recalling their child’s early reading sessions). Some parents also personified the robot in joking ways among themselves.

In summary, Emotional Attachment was a major factor keeping the robot in these homes. Even as regular use waned, families cared about the robot’s “wellbeing” and maintained a bond. This finding directly addresses why the robot stayed: it stayed because, to the families, it was *not just a device*. It had crossed into the social/emotional domain of the household. As Sung’s work suggested, seeing a robot “with affection instead of a mere object” is key to long-term interaction (Sung et al., 2010), and our participants indeed exhibited affectionate regard.

### 4.2 Symbolic value and memory preservation (RQ2)

The second major theme is that the robot held significant symbolic value for the families–as a keepsake of the child’s early years, a trophy of participation in an innovative study, or a representation of the family’s values and identity. Eleven families emphasized symbolic value, such as preserving the robot due to its association with childhood memories, family routines, or a specific developmental stage. This theme is most closely aligned with RQ2 (emotional and symbolic value of the robot in the long term). We detail sub-aspects of this symbolic retention:

• Keepsake of Childhood (“Memory Box” Object): Nearly every parent explicitly mentioned that the robot reminds them of the child’s younger days. One parent described the robot as “freeze-framing a part of her childhood” (Family 7). Others echoed this sentiment, likening it to a baby book or favorite blanket–something emotionally irreplaceable. For these parents, giving away the robot would be like throwing away a baby book or a favorite blanket–unthinkable from a sentimental perspective. Parents and children sometimes joked about the robot’s future presence in college or beyond, underscoring its symbolic resonance. The mother in Family 15 followed up more seriously, *“It symbolizes how much she’s grown. She does not need it to read now, but it is a reminder of how she learned.”*
• Milestone Marker and Achievement Symbol: Some families saw the robot as a symbol of the child’s learning achievements. One family called it a “trophy of how far [their child] has come,” proudly displaying the robot and associated books as a marker of growth. Similarly, Family 12s parent noted that the robot itself became a conversational piece to talk about the child’s reading journey: *“We sometimes show visitors: ‘Here’s the little guy that taught Ben to read!’ It is a great story to tell.”* In this way, the conversion function (Be et al., 2005) is evident–the robot became part of the family narrative and something they take pride in showcasing.
• Conversation Piece/Tech Showcase: About half the families mentioned that visitors or friends find the robot interesting, and this reinforced their desire to keep it. Families appreciated having a unique or “cool” object in the home, sometimes calling it their “family mascot.” This uniqueness enhanced the robot’s value as a conversation piece or tech symbol.
• Transitional Object and Emotional Symbolism: Several parents and children drew analogies to other cherished objects. One mother (Family 11) compared the robot to her daughter’s old blanket. The child, Mira, kept the robot on the same shelf as storybooks and special toys, saying: *“I do not play with them all the time, but I like knowing they’re there.”* This suggests that the robot, like a transitional object, remained emotionally significant even after active use diminished.
• Family Identity and Values: In a few interviews, parents talked about how keeping the robot aligns with their family values. For example, one parent said: *“We’re a family that does not give up on things…The robot is part of our history.*” Another described it as an “icon” of learning. These comments show that for some, the robot turned into an artifact of family culture–symbolizing aspects like perseverance, love of learning, or openness to innovation.

In summary, the Symbolic Retention theme shows that the robot served as a cherished artifact—representing early childhood, family identity, and learning milestones.

### 4.3 Practical repurposing and daily integration (RQ3)

The third theme pertains to how families integrated the robot into their household practices over time, often by repurposing it for functions beyond the original reading tutor role or by incorporating it into new routines. Fourteen families engaged in routines that integrated the robot into their household practices—such as bedtime rituals, display decisions, or use in younger siblings’ play—extending its perceived usefulness beyond its original purpose. This theme responds directly to RQ3 (household practices and ongoing integration). This theme explains how the robot continued to be used (albeit at lower intensity) and how it physically and functionally fit into home life in 2025.

**FIGURE 3:**
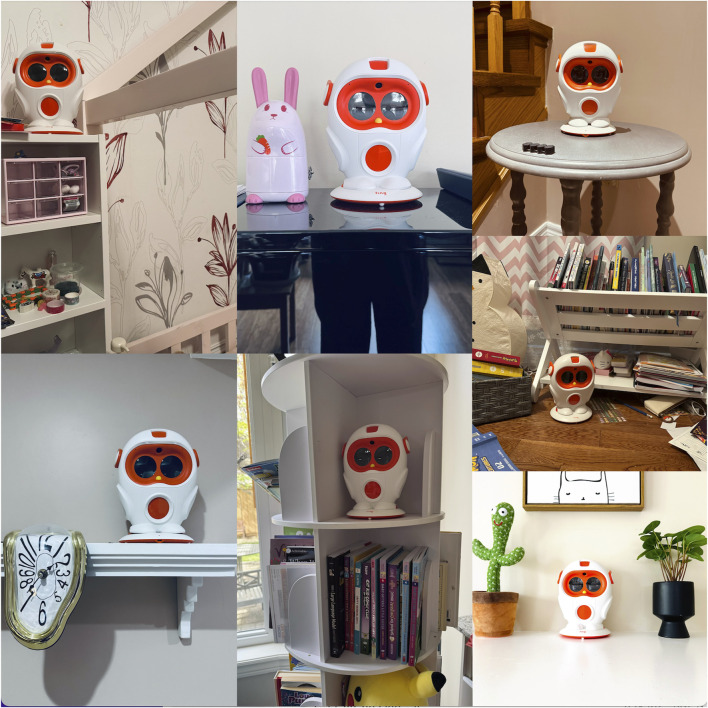
Photographs showing various placements of the retired robot in participants’ homes. These images illustrate how families continued to display the robot in visible, meaningful locations such as shelves, desks, and common areas, often alongside other cherished objects, signaling its lasting presence in household routines and family identity.

Key aspects include:

• Storytelling and Entertainment: About half families (10 out of 19) reported that they *still use the robot occasionally as a storyteller or entertainer.* Families discovered that the robot could still entertain older children or siblings through stories or jokes, often using it during family time. Some older children used the robot with younger siblings, passing on its use as a shared activity, especially for bedtime stories. Repurposing often involved handing down the robot to younger siblings, extending its educational and entertainment life. In some cases, older children incorporated the robot into imaginative play, using it as a prop or responsive character. This inventive use underscores the robot’s flexibility as an interactive object in play, contributing to its continued relevance.
• Music: Several families leveraged the robot’s ability to play music or make sounds. Children enjoyed using the robot for music and playful eye animations, adding to its appeal even after outgrowing its reading function.
• Ambient Presence and Alert Functions: A few tech-savvy parents tinkered with the robot to integrate it into their smart home. Some parents programmed the robot for simple greetings or reminders, giving it a functional role in household routines. One family used the robot as a chore reminder—its voice sometimes proving more effective than a parent’s. These are examples of incorporation into household routines–the robot had specific places and times when it was used, even if those were occasional (like greeting or holiday entertainment).
• Maintenance Rituals: Even when not actively “used” for play or tasks, many families had routine practices around *maintaining* the robot, which effectively integrated it into their domestic routines. Charging the robot regularly became a ritual in some homes, akin to *“watering a plant*,” as one parent described. A few families attempted to update the robot’s software, even after support ended, underscoring their continued interest and emotional investment. This resonates with how AIBO owners reacted when Sony ended support (Burch, 2018). In our case, fortunately, the robot’s basic functions did not depend on external servers, so families could continue offline use.
• Physical Placement and Display: Integration was also physical. Most families kept the robot in a visible, accessible location, reinforcing its presence in daily life. It was not stuffed in a closet (except for the one relinquished case). Children often displayed the robot with books or toys, reflecting its emotional and symbolic value. The placement often signified the robot’s *role.* On bookshelves or study desks, it was like a guardian of knowledge or a bookend to the child’s reading journey. In living rooms, it was treated more as a decor or tech showcase. A few families even decorated around the robot: Family 3 had a small doily underneath it (“like an accent piece,” the mom said with a chuckle), and Family 15 hung a little name plaque above it that the child had crafted. These touches show the robot being *objectified* in the home space intentionally, blending with interior decor. Figure 3 presents examples of how participants positioned Luka in their homes.

One subtheme to highlight is the role transition the robot underwent in many families. Initially, it was an authority figure (a teacher/tutor). By 2025, it often became either a peer-like companion or a subordinate role (like a toy or tool controlled by the child). For example, *Emma (8 yrs., Family 19)* said: *“I’m the teacher now and Luka is my student sometimes. I teach him new stories.”* This indicates how the child’s perspective shifted–she no longer looked up to the robot for knowledge, but rather used the robot as a prop to enact her own mastery of reading. Similarly, siblings used the robot as an assistant (reading to younger kids). In some families, parents became the primary “users” by using it for home automation or as a music player. This fluidity of role demonstrates the robot’s versatility but also the necessity of that versatility for its survival. Had it rigidly been only a reading tutor, it likely would have been shelved. Instead, families found these new roles: entertainer, pet, decoration, reminder, *etc.* A visual way to represent this is a flow from “Tutor” (2021) to “Friend/Pet”, “Keepsake”, “Tool/Device” (2025). We could imagine a diagram of these role transitions, similar to what we presented conceptually in Figure 3 (where “Repurposed Usage” and “Physical Presence” nodes indicate new roles).

Finally, it is notable that only one family (Family 15) no longer had the robot by 2025. In that case, the family’s circumstances were unique: they moved to a smaller apartment in 2023 and decided to declutter. The mother said it was a hard decision, but her daughter had *“pretty much stopped using it entirely”* by age 7, and they knew a cousin’s younger child who could benefit from it.

In summary, Practical Repurposing and Integration explains how the robot continued to have *functional presence*, even if minimal, in family life. Families adapted the robot to new uses (storytelling, music, companion for siblings), maintained it as a powered and present device, and physically included it in their living spaces. These practices reinforced the robot’s persistence: it was easier to justify keeping something that still did *something*, however small. The thematic evidence suggests that while emotional and symbolic factors provided the motivation to keep the robot, these practical integrations provided the means to occasionally engage with it and keep it from becoming “just a dead object.” The robot, in essence, was *domesticated*–it found a stable, if evolving, niche in each home’s ecology.

### 4.4 Leeting go: a divergent case (RQ1, RQ2)

This exception case helps contextualize RQ1 and RQ2 by illustrating a contrasting scenario where emotional and symbolic attachment were less prominent. As noted, 18 of 19 follow-up families retained the robot. The single family that did not (Family 15, introduced above) offers a useful counterpoint to highlight what might lead to a robot’s removal. In that case, the family’s attachment to the robot was relatively lower and the practical use had ceased completely by around 2 years post-deployment. In their interview, the mother said: *“After she started first grade, she did not use it at all. We kept it another year or so, mostly because I felt bad to just throw it out and it looked cute in her room. But when we had to downsize, we asked if she was okay giving it to her younger cousin and she was.”* The daughter (then 9) did not object much, saying she “outgrew” it and was more into her tablet and other toys by then.

Why was their attachment lower? The mother speculated: *“Maybe because she always saw it more as a tool than a friend. She liked it, but she was not as into pretending it was alive.”* Indeed, this child, according to the mother, was a bit more mature in her thinking even at five and viewed the robot like a talking appliance. So anthropomorphism was less, and once the function was done, she moved on. This aligns with Kühne et al.‘s finding that some children have a lower anthropomorphism trajectory (Kühne et al., 2024). Furthermore, the family had many other keepsakes from childhood (the mom mentioned they kept a lot of artwork and were “swamped with stuff”), so the robot was not uniquely cherished as a memory object compared to others.

Interestingly, even for this family, the decision was to re-home the robot to a relative rather than discard it. This suggests a sense of the robot’s value and perhaps residual attachment (the mom admitted *“I’m glad it is with my nephew; I did not want it in a landfill”*). In its new home (we did not formally interview the cousin’s family, but heard anecdotally), the robot was reportedly doing well as a reading buddy again. In a way, this outcome is a successful “retirement” where the robot gets a second life. It also raises a point: none of our families reported the robot breaking down completely. Had it broken, perhaps we’d have seen an emotional reaction or attempts to repair [like AIBO owners do (Burch, 2018)]. The hardware’s durability likely helped–it remained as a tangible object to care for or repurpose.

This counterpoint case reinforces the main themes by contrast. It lacked strong attachment (Theme 4.1), and the family did not heavily integrate or repurpose the robot beyond its initial use (Theme 4.3). Once the symbolic value was not sufficient, they parted with it–but did so in a considerate manner that still acknowledged the robot’s quasi-agent status (they “gave” it to someone who would interact with it, akin to a good adoption).

Overall, our results demonstrate that the persistence of the robot in the other 18 families was underpinned by a combination of emotional bonds, symbolic importance, and adaptive household integration. Even as active educational use dwindled, the robot remained a valued presence–whether as a beloved friend, a trophy of growth, or a quirky smart appliance. In the next section, we discuss these findings in a broader context, examining what they mean for designing future social robots and understanding the life cycle of companion technologies in homes.

## 5 Discussion

Our follow-up study reveals a multifaceted picture of how families engaged with a social robot 4 years after its introduction as a preschool reading tutor. The robot’s journey from a daily learning companion to a “retired” household member provides rich insights into long-term human-robot relationships. In this Discussion, we interpret our findings in light of prior research and theoretical frameworks, discuss implications for social robot design and HRI theory, and reflect on the broader cultural meaning of retaining robots beyond their period of functional use.

Our findings address the three research questions as follows: RQ1 is answered through evidence of shifting perceptions—from educational tool to companion, pet, or symbolic object—as detailed in Sections 4.1 and 4.4. RQ2 is addressed through families’ deep emotional attachment, symbolic framing, and preservation behaviors (Section 4.2), demonstrating that the robot’s perceived value extended well beyond its educational purpose. Finally, RQ3 is answered by the multiple ways families repurposed and integrated the robot into daily life, including maintenance rituals, smart home adaptations, and sibling use (Section 4.3). These results demonstrate that retention was not merely passive but actively maintained through emotional, symbolic, and practical routines.

### 5.1 Emotional attachment as a driver of longevity

For many families, emotional attachment appeared to play a meaningful role in the robot’s continued presence in the home. Twelve families described strong affection or personification, suggesting that such bonds may have contributed to its preservation and use over time. Families that kept the robot typically developed affective bonds with it, treating it with care and concern much like a pet or cherished toy. As described in Section 4.1, families such as Family three and Family eight demonstrated strong emotional bonds through personification and care behaviors. This aligns with the notion that endowing a robot with social presence and engaging personality can foster long-term use (Leite et al., 2013). In our study, children continued to attribute human-like qualities (“he gets lonely,” “he’s like my brother”) well beyond the initial novelty phase. Such anthropomorphic and empathetic perceptions made the idea of discarding the robot emotionally aversive. This aligns with the patterns reported in Section 4.2, where children expressed concern about the robot’s feelings or wellbeing.

This dynamic can be understood through the lens of attachment theory. Children in 10 of the 19 families, and occasionally parents, appeared to form an attachment to the robot as a transitional object or attachment figure. The robot often provided comfort and a sense of companionship, which are hallmarks of attachment relationships. For example, in Family 15, the child viewed the robot more as a talking appliance, and they were the only family to part with it. These variations in attachment were clearly reflected in the contrasting experiences of Family 3 (strong emotional bond) and Family 15 (utilitarian perception), as outlined in Sections 4.1 and 4.4. This variation resonates with Kühne et al.‘s finding that some children maintain high anthropomorphism and others low over time (Kühne et al., 2024). Design-wise, this suggests that building in features that nurture emotional engagement (e.g., personalization, expressing “needs” like charging in a pet-like way) could increase the proportion of users who develop sustained attachment. Our data showed that families who felt the robot needed their care (like keeping it charged) were very likely to keep it around.

The phenomenon observed parallels what happens with robot pets like AIBO and PARO. People project life onto these robots and thus include them in their social sphere (Burch, 2018). A key difference is that our robot started as a *tool for education*, not explicitly a pet. Yet over time, families effectively *re-framed* it as a pseudo-pet or friend. This re-framing was crucial–it allowed the robot to transition out of its job description and still matter. It is reminiscent of how some working animals (like a guide dog) may be kept as a pet after retirement due to emotional bonds. In HRI terms, the robot’s role shifted from task-oriented to relationship-oriented.

For HRI researchers and designers, this underscores the importance of designing for relationship longevity. If we desire robots to be more than short-term gadgets, fostering attachment is important. However, we must also consider ethical implications: users (especially children) might feel distress when a robot “dies” or is removed (Burch, 2018). In our study, one mother noted her son cried when the robot’s battery died, indicating a level of emotional vulnerability. Designers might need to implement “graceful” end-of-life mechanisms or support features for long-term robots. For example, providing a way to back up a robot’s persona or transfer it to a new device could ease the eventual transition (akin to how some people wanted to transfer AIBO’s “soul” to a new unit). While our study’s timeframe did not yet force such decisions (the robots were still operational), it foreshadows that as products age, emotional attachment can become a double-edged sword if the robot cannot be maintained indefinitely.

### 5.2 The domestic life cycle: from novelty to normalcy to nostalgia

The evolution of the robot’s role in the families reflects a domestic life cycle that can be mapped onto domestication theory phases. Fifteen families retrospectively described the robot’s role as evolving from novelty to normalization to nostalgia. While this suggests a trajectory, we caution that this framing is based on recall rather than longitudinal observation. Initially, the robot was appropriated as an educational tool (with excitement and novelty). Over time, it became incorporated into routines (e.g., nightly reading, playtime), then reached a phase of normalization [children no longer found it novel by year 2, akin to Kanda et al.‘s observation of kids seeing the robot as part of the class (Kahn et al., 2012)]. Finally, by year 4, the robot often held a place of identification/nostalgia–it was not strongly functional but was kept for what it meant. In some sense, according to retrospective accounts, the robot became *invisible yet present*, similar to Tanaka’s finding of robots becoming background actors that are still socially significant (Tanaka et al., 2007).

Our results highlight a stage that is not often discussed: the post-functional retention stage, where a device is kept primarily for sentimental or symbolic reasons. This might be seen as an extension of domestication theory’s *conversion* phase: the technology’s meaning (to users and how it is presented to others) overtakes its practical use. Several families - particularly Families 1, 6, and 17 - displayed the robot as a proud reminder of childhood or using it as a conversation piece exemplify this. In technology adoption literature, there’s an analogue in how people keep old phones for nostalgia or collectors keep outdated tech because of its meaning (Alizadeh et al., 2022). For social robots, this stage is arguably more pronounced because of their social nature–people feel they ought to treat the robot “respectfully” even after retirement.

Another way to frame this is through the concept of a technology’s social life. Our robot’s social life did not end when its job ended; it transitioned roles. This is reminiscent of research on how products can be reimagined by users (Wakkary et al., talk about “things we could design for the afterlife of products”) (Wakkary, 2021). For robotics, this might mean designing robots that can gracefully shift roles as user needs change. For example, an educational robot might intentionally have secondary modes (entertainment, companion) that become prominent as the education mode becomes less relevant. In our study, families found those secondary uses on their own (like using it as a music player). Designers could support that better by including growth content or adaptive functionality that matures with the child. Kory-Westlund and Breazeal attempted something similar by having a robot adapt stories to a child over a long term (Park et al., 2020). One can imagine an “aging with the child” design: the robot’s persona could evolve from teacher to peer to junior (flipping the script so the child teaches the robot something as they grow). Some children spontaneously did this, which suggests designing features to encourage such play could harness developmental changes rather than suffer from them. For example, Emma (Family 19) said, *‘I’m the teacher now and Luka is my student,’* reflecting a developmental re-framing that extended the robot’s relevance beyond its initial instructional role (see Section 4.3).

Importantly, active use was not necessary for the robot to remain a meaningful part of domestic life. This implies that metrics of success for long-term robots should not rely solely on usage frequency. A robot might be turned on only a few times a month but still be valued and therefore retained. Traditional human-computer interaction metrics would flag low usage as failure, but in our qualitative context, low usage did not equal abandonment. Instead, *the robot moved from foreground to background*. This is akin to how a once-favorite toy might sit on a shelf but still be loved.

### 5.3 Design implications: designing robots with a life cycle in mind

Our findings yield several concrete design implications for social robots intended for long-term use in homes:

1. Plan for Role Transitions: Robots should be designed with the expectation that their primary role for a user will evolve. Rather than a single use-case, include multiple modes or features that can become relevant at different life stages or to different family members. For instance, an educational robot could also have a library of jokes, trivia, or games that an older child can enjoy. It could have a “companion mode” where it engages in free-form conversation or listens, which might be more appreciated as the child grows (even if just as a novelty). By *designing for versatility*, we give the robot a better chance to remain useful or at least occasionally engaging. Our study saw organic repurposing; design could make that easier (e.g., a straightforward way to switch to an entertainment mode labeled “For when I’m older!” in the interface).
2. Support Emotional Continuity: If a robot is meant to be kept for years, its design should consider how to maintain the emotional relationship. This might involve the robot remembering past interactions and bringing them up later (“I remember when you used to read me *Cat in the Hat*, that was fun!“). Such callbacks could reinforce the nostalgic value and signal to the user that the robot shares memories–deepening its identity as a companion through time. We saw children doing this reminiscing themselves; a robot that actively participates in reminiscing could be powerful in cementing long-term bonding. However, designers should do this carefully to avoid dissonance if the child’s attitudes change.
3. Gradual Autonomy or Dependence Shift: Early on, the robot was something the child depended on; later it became something that depended on the family’s care (for power, *etc.*). Designers might consider an intentional shift in how the robot presents itself. For example, as the child grows, the robot could start expressing more “needs” (like please charge me) in a way that invites the child to take on a caregiving role (flipping the dynamic). This could leverage children’s nurturing instincts (similar to a Tamagotchi or virtual pet) to keep them engaged in caring for the robot even if they no longer need its lessons. One could also do the opposite: early on the robot is needy (promoting bonding), then later it becomes more autonomous to avoid burdening an older child–but our data suggests older kids *liked* caring for it to some extent, so leaning into the caregiving might work well.
4. Durability and Support for the Long Haul: On a practical level, for a robot to survive as long as attachment lasts, it needs to be durable and supported. Families in our study expressed dismay at the idea of the robot losing software support or breaking. One family noted concern when updates stopped, saying the robot felt ‘cut off,’ and they were relieved that basic functions still worked offline (Section 4.3). This concern underscores how continued functionality was tied to the robot’s perceived ‘aliveness’ and influenced decisions to maintain it. Alternatively, releasing update packages or open-sourcing some software when a product is discontinued could help devoted users keep it alive. Given how strongly people can feel about “betrayal” when a beloved device is bricked, it is an ethical consideration for companies [as also argued by Sciuto et al. (2018) regarding IoT devices’ longevity] (Sciuto et al., 2018). Our families did not have a full Jibo-like shutdown scenario, but if they did, we suspect many would react with frustration or grief.
5. Exit Strategies and Handover Rituals: Designing for the end-of-life is as important as designing for continued use. Our findings on the one family who re-homed the robot show the value of a *positive narrative* around parting with the robot (e.g., framing it as the robot going to help another child). Designers and researchers might consider creating guidance for “retiring” a robot in healthy ways. Perhaps the robot itself could facilitate this if it senses it is being used less–e.g., suggesting “I can be donated to a library or school to help other kids” in a cheerful way. This might sound fanciful, but it could alleviate guilt and give closure. It treats the robot almost like a character that can bow out gracefully. There is some precedent: an AI or game character sometimes “leaves” the storyline in a satisfying way instead of just stopping. In human-robot attachment literature, there are calls for developing protocols for robot end-of-life (​​ et al.). Our real-world data strongly supports that need–families effectively improvised a ritual (charging it one last time, explaining to the child, *etc.*). Designers could formalize or assist that.

### 5.4 Children growing up with robots: developmental reflections

From a developmental standpoint, our study suggests that children can maintain nuanced relationships with robots as they grow. Early on, imagination and belief drive engagement; later, nostalgia and affection take over, even if the belief in the robot’s aliveness diminishes. This shows children’s remarkable capacity for what could be called “suspension of disbelief” or dual reality–they know the robot is not alive, yet they act kindly toward it. This is similar to older kids still being gentle with dolls or stuffed animals (knowing they’re not real but treating them as if they have feelings out of empathy). It challenges any notion that once children “know the truth” about a robot, they will discard it. Human relationships with fictional or non-living entities (like imaginary friends, or even virtual game characters) often persist beyond factual understanding. Among the families in our study, ten children described the robot in ways that suggest a shift from initial fascination to a deeper, more personal fondness over time. While this trajectory was not universal, several narratives indicated that the robot’s role evolved as children matured, becoming less about novelty and more about emotional resonance.

Moreover, involving children in follow-up reflections about the robot provided insight into their perspective on how they’ve changed. Some kids expressed pride in how the robot helped them and saw keeping it as honoring that part of themselves. This suggests that long-term HRI can have positive effects on children’s self-concept–e.g., “Luka reminds me I’m a capable reader now.” It can almost act as a mirror of their growth. Future work could delve deeper into psychological outcomes of long-term child-robot interaction, such as memory formation, self-efficacy (“I outgrew my robot, I must have learned a lot!“), *etc.* Our data offers preliminary insights into these developmental outcomes, but a focused longitudinal or developmental analysis would be needed to confirm such effects.

### 5.5 Cultural and societal meaning of retired robots

Zooming out, what does it mean that families keep robots that no longer serve their original purpose? This emerging reality may become more common as personal robots proliferate. We might see a generation that has “old robot companions” sitting in their attic much like old photo albums–artifacts of personal history. Culturally, this raises fascinating questions. For instance, will we develop social norms or rituals around robot retirement? In our small sample, we saw micro-rituals (like cleaning it regularly, giving it a name plaque, saying goodnight to it). These behaviors anthropomorphize the robot and integrate it into family rituals.

Anthropologists (Arnd-Caddigan, 2015) has argued that when people form relationships with machines, even knowing they are not alive, the relationships are real to them. They suggest that as robots become increasingly social, we must treat their “retirements” or deaths with care. Our findings support this claim: several families appeared to perform an informal social afterlife for the robot, either by keeping it visible and respected or by thoughtfully passing it on. One family mentioned they would “feel wrong” throwing it out—highlighting that, as with heirlooms or meaningful toys, robots can accumulate moral weight through prolonged cohabitation and shared history.

As social robots enter more homes, they will likely evoke similar forms of everyday ethics. Just as societies have developed environmental rituals for e-waste or data-deletion rituals for old phones and computers, we may see more deliberate robot decommissioning practices. Already, communities have emerged around defunct robots like Jibo and Cozmo (Carman, 2019), creating user-maintained software or hosting farewell events. These social phenomena highlight the affective surplus of robots—how they persist emotionally even when they no longer operate technically. In time, this could lead to institutional support for robot reuse, repair, or ceremonial retirement (e.g., donation programs, school repurposing, or museum-style curation of historical robots).

Finally, this has implications for how we frame ownership and legacy. In a world of increasingly personalized technology, robots may outlast their initial users and become intergenerational objects. One family in our study speculated their robot might 1 day be played with by future grandchildren—mirroring how cherished toys or books get passed down. Designers, educators, and policymakers should consider these symbolic and cultural dimensions of long-term robot presence. What kind of legacy should a robot leave behind? Who is responsible for it when its user outgrows it? These questions may soon become as central as those of functionality or user experience.

In short, our study suggests that the robot’s retirement is not an endpoint, but rather a transition into a new social role. In keeping it, families expressed not only affection but also a subtle recognition that the robot had participated meaningfully in their lives. As companion robots grow more common, society will need new narratives, practices, and support systems to honor what these technologies mean—even when they no longer “do” anything. The robot that stayed becomes more than a device—it becomes a story keeper, a silent witness, and a symbol of a shared past.

### 5.6 Limitations and future work

While our study offers novel insights, it has limitations. The sample size (19 families) is modest and drawn from those who participated in an initial deployment–these families were likely more motivated and tech-positive than a random household. Thus, their experiences may not generalize to all owners of social robots. Moreover, cultural factors were not deeply examined–all families were in a similar geographic region and socio-economic range. In cultures with different attitudes towards animism or technology, long-term retention might differ (e.g., some cultures might more readily ritualize the disposal of objects). Future research should explore diverse contexts, including non-Western cultures and different robot types (educational, therapeutic, domestic robots).

Another limitation is that our data relies on self-report and our interpretation; This study reflects retrospective accounts from a single follow-up point, conducted 4 years after the original deployment. As such, any references to change over time—such as the transition from novelty to normalization to nostalgia—are based on participants’ recollections rather than observed longitudinal data. While these narratives provide valuable insight into perceived developmental and relational trajectories, they may be influenced by memory bias, idealization, or selective recall. However, the consistency of narratives and the triangulation between parent and child reports give us confidence in the qualitative trends described. Still, a complementary quantitative approach (e.g., tracking actual power-on events for those 4 years) would strengthen the findings.

Future work could proceed in several directions. A longitudinal study following a cohort of robot-owning families over an even longer span (e.g., a decade) would be invaluable to see if the patterns hold as children enter adolescence. Will the robots remain cherished into the teenage years, or eventually be boxed up? Additionally, studies on *discontinued commercial robots* (like Jibo or Cozmo) could reveal how everyday consumers handle robot retirement–do they keep the device, repurpose it, or feel emotional distress when servers shut down? Comparing those real-world cases to our research context can validate and extend our conclusions.

Investigating the psychological effects on children is another avenue. Does keeping a childhood robot provide comfort during transitions (like entering middle school)? Does it contribute to a child’s emotional resilience or continuity of identity? There could be therapeutic value in transitional objects like robots that future child-robot interaction research can tap into.

On the design side, future work can involve participatory design with families to envision features that would make a robot more endearing long-term or easier to let go of when the time comes. Also, exploring how to mark the end of a robot’s life in a healthy way (perhaps through a “farewell protocol” or repurposing into a non-interactive keepsake mode) could bridge HRI and thanato-technology (technology for end-of-life).

## 6 Conclusion

This study followed 19 families who hosted a social robot over 4 years, uncovering how and why a robot can remain present in a home after its original purpose has been outgrown. Our findings demonstrate that families often transform a functional device into a beloved companion and symbolic artifact. Emotional attachment and anthropomorphic care, coupled with creative repurposing and deep symbolic meaning, allowed “the robot that stayed” to become part of the family fabric. In essence, the robot graduated from teacher to friend, from utility to heirloom.

These insights contribute to the HRI literature by illuminating the *post-adoption* phase of the technology lifecycle–a phase rich with social and emotional complexity that is rarely studied. We showed that long-term acceptance is not merely about continued task-use; it is about the integration of a robot into the story of people’s lives. For designers and researchers, this underscores the importance of designing robots with lifecycle in mind–supporting not only active use but also graceful aging, sustained relationships, and meaningful goodbyes.

As social robots become more common in homes, we may witness a future where households have generations of robots: some active, some retired but kept in places of honor. The way children and parents in our study talked about their robot–with empathy, humor, and respect–gives a hopeful perspective on a future of human-robot cohabitation. Robots, if designed and adopted right, can transcend their algorithms and become something more to us: partners in our personal histories. Our research offers a glimpse of that future, one where the end of a robot’s functional life is not the end of its importance. In that sense, “the robot that stayed” teaches us not just about technology design, but about human attachment, adaptation, and the enduring capacity to find meaning in the tools that shape our lives.

## Data Availability

The raw data supporting the conclusions of this article will be made available by the authors, without undue reservation.

## References

[B1] Alizadeh F. Mniestri A. Uhde A. Stevens G. (2022). “On appropriation and nostalgic reminiscence of technology,” in Extended abstracts of the 2022 CHI conference on human factors in computing systems (CHI EA ’22) (New York, NY: Association for Computing Machinery), 1–6. 10.1145/3491101.3519676

[B2] Arnd-Caddigan M. (2015). Sherry Turkle: alone together: why we expect more from technology and less from each other. New York: Basic Books, 348.

[B3] Bender J. (2023). 40% of adults still sleep with a stuffed animal. Here’s Why. Available online at: https://www.sleep.com/sleep-tech/stuffed-animals-for-adults#:∼:text=40,transition%20from%20dependence%20to (Accessed March 2, 2025).

[B4] Bentley F. Luvogt C. Silverman M. Wirasinghe R. White B. Lottridge D. (2018). Understanding the long-term use of smart speaker assistants. Proc. ACM Interact. Mob. Wearable Ubiquitous Technol. 2 (3), 1–24. 10.1145/3264901

[B5] Berker T. Hartmann M. Punie Y. Ward K. (2005). Domestication of media and technology. UK: McGraw-Hill Education.

[B6] Broadbent E. (2017). Interactions with robots: the truths we reveal about ourselves. Annu. Rev. Psychol. 68 (1), 627–652. 10.1146/annurev-psych-010416-043958 27648986

[B7] Burch J. (2018). AIBO robot dogs given buddhist funeral in Japan. Available online at: https://www.nationalgeographic.com/travel/article/in-japan--a-buddhist-funeral-service-for-robot-dogs#:∼:text=Over%20time%2C%20they%20would%20come,helped%20build%20the%20modern%20world (Accessed March 2, 2025).

[B8] Cagiltay B. Mutlu B. Kerr M. L. (2023). “Family theories in child-robot interactions: understanding families as a whole for child-robot interaction design,” in Proceedings of the 22nd annual ACM interaction design and children conference (IDC ’23) (New York, NY: Association for Computing Machinery), 367–374. 10.1145/3585088.3589386

[B9] Carman A. (2019). They welcomed a robot into their family, now they’re mourning its death. Available online at: https://www.theverge.com/2019/6/19/18682780/jibo-death-server-update-social-robot-mourning (Accessed March 2, 2025).

[B10] Chen H. Kim Y. Patterson K. Breazeal C. Park H. W. (2025). Social robots as conversational catalysts: enhancing long-term human-human interaction at home. Sci. Robotics 10 (100), eadk3307. 10.1126/scirobotics.adk3307 40073083

[B12] Clarke V. Braun V. (2017). Thematic analysis. J. Posit. Psychol. 12 (3), 297–298. 10.1080/17439760.2016.1262613

[B13] Coggins T. N. (2022). More work for Roomba? Domestic robots, housework and the production of privacy. Prometheus 38 (1), 98–112. 10.13169/prometheus.38.1.0098

[B14] De Graaf M. M. Allouch S. B. Klamer T. (2015). Sharing a life with Harvey: exploring the acceptance of and relationship-building with a social robot. Comput. Hum. Behav. 43, 1–14. 10.1016/j.chb.2014.10.030

[B15] De Graaf M. M. Ben Allouch S. Van Dijk J. A. (2016). Long-term evaluation of a social robot in real homes. Interact. Stud. 17 (3), 461–490. 10.1075/is.17.3.08deg

[B16] De Graaf M. M. Ben Allouch S. Van Dijk J. A. (2019). Why would I use this in my home? A model of domestic social robot acceptance. Human–Computer Interact. 34 (2), 115–173. 10.1080/07370024.2017.1312406

[B17] De Visser E. J. Monfort S. S. Goodyear K. Lu L. O’Hara M. Lee M. R. (2017). A little anthropomorphism goes a long way: effects of oxytocin on trust, compliance, and team performance with automated agents. Hum. Factors 59 (1), 116–133. 10.1177/0018720816687205 28146673 PMC5477060

[B18] Donnermann M. Schaper P. Lugrin B. (2022). Social robots in applied settings: a long-term study on adaptive robotic tutors in higher education. Front. Robotics AI 9, 831633. 10.3389/frobt.2022.831633 PMC8964977 35368432

[B19] Fereday J. Muir-Cochrane E. (2006). Demonstrating rigor using thematic analysis: a hybrid approach of inductive and deductive coding and theme development. Int. J. Qual. Methods 5 (1), 80–92. 10.1177/160940690600500107

[B21] Forlizzi J. DiSalvo C. (2006). “Service robots in the domestic environment: a study of the roomba vacuum in the home,” in Proceedings of the 1st ACM SIGCHI/SIGART conference on Human-robot interaction (HRI ’06) (New York, NY: Association for Computing Machinery), 258–265. 10.1145/1121241.1121286

[B22] Geva N. Hermoni N. Levy-Tzedek S. (2022). Interaction matters: the effect of touching the social robot PARO on pain and stress is stronger when turned ON vs. OFF. Front. Robotics AI 9, 926185. 10.3389/frobt.2022.926185 PMC9305613 35875704

[B23] Kahn P. H., Jr. Kanda T. Ishiguro H. Freier N. G. Severson R. L. Gill B. T. (2012). “Robovie, you'll have to go into the closet now”: children's social and moral relationships with a humanoid robot. Dev. Psychol. 48 (2), 303–314. 10.1037/a0027033 22369338

[B24] Kanda T. Sato R. Saiwaki N. Ishiguro H. (2007). A two-month field trial in an elementary school for long-term human–robot interaction. IEEE Trans. Robotics 23 (5), 962–971. 10.1109/tro.2007.904904

[B25] Kühne R. Peter J. de Jong C. Barco A. (2024). How does children’s anthropomorphism of a social robot develop over time? A six-wave panel study. Int. J. Soc. Robotics 16 (7), 1665–1679. 10.1007/s12369-024-01155-9

[B26] Lashbrook A. (2021). The people who treat their roombas like family. Available online at: https://debugger.medium.com/the-people-who-treat-their-roombas-like-family-dee5bc74dd23 (Accessed March 2, 2025).

[B27] Leite I. Martinho C. Paiva A. (2013). Social robots for long-term interaction: a survey. Int. J. Soc. Robotics 5, 291–308. 10.1007/s12369-013-0178-y

[B28] Lupieri S. (2024). AI chatbots, smartphones, robots: our “digital teddy bears” carry A dark side. Available online at: https://worldcrunch.com/tech-science/smartphones-digital-teddy-bears/#:∼:text=transfer,And%20we%20don%E2%80%99t%20deprive%20ourselves (Accessed March 2, 2025).

[B29] Michaelis J. E. Mutlu B. (2017). “Someone to read with: design of and experiences with an in-home learning companion robot for reading,” in Proceedings of the 2017 CHI conference on human factors in computing systems (CHI ’17) (New York, NY: Association for Computing Machinery), 301–312. 10.1145/3025453.3025499

[B30] Michaelis J. E. Mutlu B. (2018). Reading socially: transforming the in-home reading experience with a learning-companion robot. Sci. Robotics 3 (21), eaat5999. 10.1126/scirobotics.aat5999 33141721

[B31] Miller D. (2008). The comfort of things. Cambridge, United Kingdom: Polity Press.

[B32] Park C. H. Ros R. Kwak S. S. Huang C. M. Lemaignan S. (2020). Editorial: towards real world impacts: design, development, and deployment of social robots in the wild. Front. Robotics AI 7, 600830. 10.3389/frobt.2020.600830 PMC7805884 33501361

[B33] Sciuto A. Saini A. Forlizzi J. Hong J. I. (2018). “Hey Alexa, what’s up?” A mixed-methods studies of in-home conversational agent usage,” in Proceedings of the 2018 designing interactive systems conference (DIS ’18) (New York, NY: Association for Computing Machinery), 857–868. 10.1145/3196709.3196772

[B34] Severinson-Eklundh K. Green A. Hüttenrauch H. (2003). Social and collaborative aspects of interaction with a service robot. Robotics Aut. Syst. 42 (3-4), 223–234. 10.1016/s0921-8890(02)00377-9

[B35] Silverstone R. (2006). “Domesticating domestication: reflections on the life of a concept,” in Domestication of media and technology. Editors Berker T. Hartmann M. Punie Y. Ward K. J. (Maidenhead, United Kingdom: Open University Press/McGraw‑Hill Education, 229.

[B36] Silverstone R. Haddon L. (1996). “Design and the domestication of information and communication technologies: Technical change and everyday life”in Communication by design: the politics of information and communication technologies. Editors Silverstone R. Mansell R. (Oxford, United Kingdom: Oxford University Press), 44, 74. 10.1093/oso/9780198289418.003.0003

[B37] Sung J. Grinter R. E. Christensen H. I. (2010). Domestic robot ecology: an initial framework to unpack long-term acceptance of robots at home. Int. J. Soc. Robotics 2, 417–429. 10.1007/s12369-010-0065-8

[B38] Tanaka F. Cicourel A. Movellan J. R. (2007). Socialization between toddlers and robots at an early childhood education center. Proc. Natl. Acad. Sci. U. S. A. 104 (46), 17954–17958. 10.1073/pnas.0707769104 17984068 PMC2084278

[B39] Veronesi C. Trimarco B. Botticelli N. Armani G. Bentenuto A. Fioriello F. (2023). Use of the PARO robot as a social mediator in a sample of children with neurodevelopmental disorders and typical development. La Clin. Ter. 174 (2), 132–138. 10.7417/CT.2023.2509 36920129

[B40] Wakkary R. (2021). Things we could design: for more than human-centered worlds. Cambridge, MA: MIT press. 10.7551/mitpress/13649.001.0001

[B41] Winnicott D. W. (1951). Transitional objects and transitional phenomena. London: Tavistock.

[B42] Yang L. Neustaedter C. (2018). Our house: living long distance with a telepresence robot. Proc. ACM Human-Computer Interact. 2 (CSCW), 1–18. 10.1145/3274459

[B43] Zhao Z. McEwen R. (2022). “Let’s read a book together”: a long-term study on the usage of pre-school children with their home companion robot,” in Proceedings of the 2022 ACM/IEEE international conference on human‑robot interaction (HRI ’22) (Sapporo, Japan: IEEE Press), 24–32. 10.1109/HRI53351.2022.9889672

